# Esophageal cancer in the elderly: an analysis of the factors associated with treatment decisions and outcomes

**DOI:** 10.1186/1471-2407-10-510

**Published:** 2010-09-24

**Authors:** David Tougeron, Hadji Hamidou, Michel Scotté, Frédéric Di Fiore, Michel Antonietti, Bernard Paillot, Pierre Michel

**Affiliations:** 1 Digestive Oncology Unit, Department of Gastroenterology, Rouen University Hospital, Northwest Cancéropôle, 1 rue de Germont, 76031 Rouen Cedex, France; 2 Department of Radiotherapy, CRLCC Becquerel, Northwest Cancéropôle, rue d'Amiens, 76000 Rouen, France; 3 Department of Digestive Surgery, Rouen University Hospital, Northwest Cancéropôle, 1 rue de Germont, 76031 Rouen Cedex, France

## Abstract

**Background:**

Only limited data has been reported so far regarding oesophageal cancer (EC) in elderly patients. The aim of the study is to identify the baseline parameters that influenced therapeutic decision.

**Methods:**

All consecutive patients 70 years or older being treated for EC were retrospectively analyzed. Patients without visceral metastasis were divided into two groups: treatment with curative intent (chemoradiotherapy, surgery, radiotherapy, mucosectomy or photodynamic therapy) or best supportive care (BSC). Patients with metastasis were divided into two groups: palliative treatment (chemotherapy, chemoradiotherapy or radiotherapy) or BSC.

**Results:**

Two hundred and eighty-two patients were studied. Mean age was 76.5 ± 5.5 years and 22.4% of patients had visceral metastasis. In patients without visceral metastasis (n = 220) the majority had treatment with curative intent (n = 151) whereas in patients with metastasis (n = 62) the majority had BSC (n = 32). Severe adverse events (≥ grade 3) were observed in only 17% of the patients. Patients without specific carcinologic treatment were older, had more weight loss, worse WHO performance status and Charlson score in multivariate analysis.

**Discussion:**

Our results suggest that elderly patients with an EC could benefit from cancer treatment without major toxicities. Weight loss, WHO performance status and the Charlson score could be used to select the appropriate treatment in an elderly patient.

## Background

In the USA, oesophageal cancer (EC) occurs in patients over 60 and 75 years of age in 44% and 30% respectively [1]. Similar data have been published in different European countries [2]. Management of elderly patients with EC remains a therapeutic challenge and the most relevant treatment modalities are still being debated. Although survival improvement has been observed over the past decade, EC treatment continues to be significantly influenced by age [3]. Moreover, it has also been reported that elderly patients have undergone less surgery, radiotherapy and chemotherapy than younger patients [4].

To our knowledge, no specific data have been published regarding therapeutic strategy in elderly patients with EC. Despite progress in surgical practice, oesophagectomy is associated with significant morbidity and mortality, and 75 years is often considered as the age limit for this type of surgery [5]. Definitive chemoradiotherapy (CRT) has been considered with curative intent in locally advanced or inoperable non-metastatic EC [6-9] but few studies have evaluated this treatment in elderly patients [10,11]. To our knowledge there is no reported data about the clinical or tumor characteristics that could influence treatment decisions in elderly patients with EC. Nevertheless, it is crucially important that clinicians have criteria for therapeutic decisions.

The aim of the study was to identify the baseline parameters that influenced both the therapeutic decision and outcomes in elderly patients with EC.

## Methods

### Patient inclusion

All patients 70 years or older with an EC hospitalized in the Gastroenterology Department of Rouen University Hospital between January 1994 and December 2007 were retrospectively evaluated. The local ethics committee approved the procedure and, due to the retrospective analysis with a majority of dead patients, no patient consent was necessary. Patient and tumor baseline characteristics were collected. Degree of dysphagia was evaluated according to the Atkinson score [12]. Charlson score based on nineteen medical conditions for the analysis of patient's comorbidities was used [13]. The tumor staging was based on the 1983 AJCC staging system according to published recommendations. Tumor TNM stage was based on esophagoscopy, barium esophagography, chest and abdominal computed tomography (CT-scan) and esophageal ultrasonography when it was feasible.

### Treatment regimen

In our institution, since the validation of definitive CRT and in accordance with the international guidelines, there was no major modification of oesophageal carcinoma therapeutic strategy during the period of the study [6,7]. The different treatment options were endoscopic resection (mucosectomy), photodynamic therapy (PDT), surgical resection, chemoradiotherapy, radiotherapy (RT) or chemotherapy (CT). For each patient, the therapeutic strategy was discussed in multidisciplinary meetings with the gastroenterologist, surgeon, oncologist and radiotherapist. The decision-making process was based not on predefined variables but with consideration of multiple doctors' opinions and on an individual basis taking into account age, albumin rate, loss of weight, comorbidities and performance status. In general, if there were several criteria including age > 75 years, albumin < 30 g/l, loss of weight > 10%, multiple severe comorbidities and WHO performance status > 1 meant that there was a contradiction for aggressive treatment (surgery, RCT or chemotherapy).

Mucosectomy and PDT were routinely reserved for *in situ *tumors (Tis) but also carried out in elderly patients with stage I oesophageal carcinoma and a contraindication for surgery (severe comorbidities) [14]. In our institution, elderly patients with stage II tumors underwent surgical resection or definitive CRT. Two operative approaches were used at the surgeon's discretion: the Ivor-Lewis transthoracic oesophagectomy with a 2-field lymphadenectomy or the transhiatal oesophagectomy with abdominal lymphadenectomy and limited mediastinal lymph node resection. Nevertheless, a majority of these patients received a CRT regimen with curative intent due to surgical contraindication. Patients with stage III tumors had definitive CRT based on the cisplatin and 5-fluorouracil (CDDP/5FU) combination as described by Herskovic *et al *[15] or the CDDP/irinotecan combination reported by Michel *et al *[16]. The radiotherapy dose was 50-55 Grays (Gy), delivered 5 days a week at 1.8 Gy/day, based on the international recommendations [6,7]. Salvage surgery could be performed in patients without clinical complete response and no metastases. Chemotherapy used in metastatic patients was principally CDDP/5FU regimen. Patients with severe malignant dysphagia and/or esorespiratory fistula and/or a contraindication for CRT or surgery (primarily severe comorbidities or a metastatic disease at diagnosis) were treated by self-expanding metallic stent (SEMS).

All severe adverse events defined as a toxicitiy grade ≥ 3 during CT (based on National Cancer Institute Common Toxicity Criteria version 2.0) or life-threatening complications after surgery or endoscopic treatment were recorded.

### Outcome and follow-up

In patients treated with curative strategy, a clinical complete response (CCR) was defined by no residual tumor on upper digestive endoscopy and no metastatic disease occurrence on CT-scan. This evaluation was performed approximately 2 months after curative treatment. In metastatic patients, chemotherapy efficacy was evaluated according to RECIST criteria. Follow-up data were updated in December 2008.

### Statistical analysis

Two independent analyses were performed according to the metastatic status of patients (stage I to III and M1a *versus *M1b). In patients without metastases two groups were compared: patients treated with curative intent (mucosectomy and PDT for T*is *or T1N0M0, surgery, CRT or RT) and those treated by best supportive care (BSC) (SEMS or any specific treatment). In metastatic patients two groups were compared: patients treated by CT, CRT or RT and those treated by BSC.

We performed an analysis of factors influencing treatment strategy in univariate and multivariate logistic regression analyses. Groups were compared using Fisher's exact test and Student's t-test as appropriate. Any variables reaching p = 0.05 were introduced in multivariate analysis.

Survival curve was established using the Kaplan-Meier method and compared with Log-rank test. Predictive factors of overall survival were studied by univariate analysis and further evaluated in multivariate Cox regression analysis to estimate the hazard ratio (HR) with a 95% confidence interval (CI). Predefined baseline variables for the univariate analysis were: sex, age ≥ 75 years, WHO performance status < 2, initial weight loss < 10%, Charlson score ≤ 1, histology, tumor stage and treatment. Any variables reaching *p *= 0.05 were introduced in multivariate analysis.

All statistical analyses were performed with a two-side significance value of 0.05. Statistical analysis was performed using the Statview software (Statview for Windows, SAS Institut Inc., version 5.0).

## Results

### Patient and tumor characteristics

**Figure 1:**
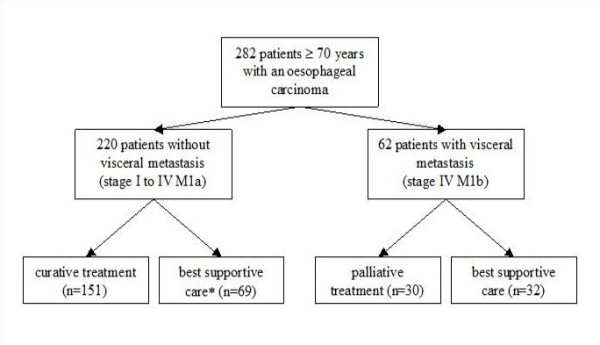
**Patient stratification**. * Two patients had a treatment with chemotherapy, others had best supportive care.

Two hundred and eighty-two patients over 70 years of age were assessed (among 904 patients with esophageal cancer). In patients without visceral metastasis (n = 220), 151 and 69 had a potential curative treatment and best supportive care respectively (Fig. 1). In patients with metastatic disease (n = 62), 32 had BSC and 30 had palliative treatment. Mean age was 76.5 ± 5.5 (range 70 to 96 years) (Table 1). The prevalence of patients with comorbidities, according to the Charlson score, was 69.3%. Fifty-five patients (21.6%) had a prior or concurrent malignancy, 45 patients (17.7%) chronic obstructive pulmonary disease, 38 patients (15.0%) myocardial infarction, 30 patients (11.8%) peripheral vascular disease, 29 patients (11.4%) diabetes and 24 patients (9.4%) congestive heart failure. The majority of patients had stage II or III tumors (Table 2).

**Table 1:** Patient characteristics

	All patients (n = 282)	Patients without visceral metastasis (n = 220)		Patients with visceral metastasis (n = 62)	
		
		Curative treatment (n = 151)	BSC (n = 69)	Palliative treatment (n = 30)	BSC (n = 32)
**Age**					
year, SD ≥ 75 years	76.5 ± 5.5	74.9 ± 4.1	80.0 ± 6.6*	74.2 ± 4.0	78.2 ± 5.8 **
	145 (51.4%)	62 (41.1%)	50 (72.5%) *	12 (40%)	21 (65.6%)
**Gender ratio** (men/women)	216/66 (76.6%)	124/27(82.1%)	38/31 (55.1%) *	27/3 (90%)	27/5 (84.3%)
					
**WHO performance status**^‡ ^(n = 260)					
0	39 (15.0%)	35 (23.3%)	0 *	3 (10.0%)	1 (3.8%) **
1	119 (45.8%)	84 (56.0%)	11 (20.4%)	18 (60.0%)	6 (23.1%)
2	93 (35.8%)	31 (20.7%)	38 (70.4%)	8 (26.7%)	16 (61.5%)
3	9 (3.5%)	0	5 (9.3%)	1 (3.3%)	3 (11.5%)
					
**Atkinson dysphagia score**^† ^(n = 270)					
0	31 (11.5%)	20 (13.2%)	7 (11.3%) *	2 (6.9%)	2 (7.1%)
1	9 (25.5%)	43 (28.5%)	6 (9.7%)	14 (48.3%)	6 (21.4%)
2	129 (47.8%)	65 (43.0%)	39 (62.9%)	10 (34.5%)	15 (53.6%)
3	30 (11.1%)	16 (10.6%)	7 (11.3%)	3 (10.3%)	4 (14.3%)
4	11 (4.1%)	7 (4.6%)	3 (4.8%)	0	1 (3.6%)
					
**Initial weight loss**^† ^(n = 219, SD)	8.4 ± 7.7	6.7 ± 6.6	12.4 ± 11.3 *	9.0 ± 5.6	13.6 ± 10.0 **
					
**Initial weight loss ≥ 10%**^†^	84 (38.4%)	40 (28.4%)	22 (64.7%) *	11 (40.7%)	11 (64.7%)
					
**Initial albumin**^† ^(n = 148, g/l, SD)	35.2 ± 6.9	37.7 ± 5.4	31.4 ± 8.0 *	35.2 ± 4.8	28.7 ± 6.2 **
					
**Creatinine clearance**^† ^(n = 172, mL/min, SD)	67.1 ± 24.5	72.4 ± 22.7	52.5 ± 23.3 *	76.1 ± 22.5	60.9 ± 26.4

**Charlson score**^†^ (n = 254)					
Mean	1.4	1.3	1.7	1.2	1.6
Charlson score at 0	78 (30.7%)	50 (38.5%)	12 (18.2%) *	8 (28.6%)	8 (26.7%)

n: number of patients, SD: standard deviation, g/l: gram per liter, mL/min: milliter per minute,

BSC: best supportive care, *: *p *< 0.05 curative treatment vs BSC, **: *p *< 0.05 palliative treatment vs BSC,

^†^: not available for all patients

**Table 2:** Tumor characteristics

	All patients (n = 282)	Patients without visceral metastasis (n = 220)		Patients with visceral metastasis (n = 62)	
		
		Curative treatment (n = 151)	BSC (n = 69)	Palliative treatment (n = 30)	BSC (n = 32)
**Tumoral stage**					
stage I	27 (9.7%)	24 (15.9%)	3 (4.3%)	-	-
stage II	68 (24.5%)	61 (40.4%)	7 (10.1%)	-	-
stage III	69 (24.9%)	51 (33.8%)	18 (26.1%)	-	-
stage IV (M1a)	8 (2.9%)	7 (4.6%)	1 (1.4%)	-	-
stage IV (M1b)	62 (22.4%)	-	-	30 (100%)	32 (100%)
unknown but M0	48 (17.0%)	8 (5.3%)	40 (58.0%)	-	-
					
**Tumoral location**					
lower third	151 (53.5%)	80 (60.0%)	37 (53.6%)	19 (63.3%)	15 (46.9%)
middle third	86 (30.6%)	44 (29.1%)	21 (30.4%)	9 (30.0%)	12 (37.5%)
upper third	45 (16.0%)	27 (17.9%)	11 (15.9%)	2 (6.7%)	5 (15.6%)
					
**Mean tumor length**^†^ (n = 199, cm, SD)	5.2 ± 2.5	4.7 ± 2.3	5.4 ± 2.6	5.9 ± 1.8	7.2 ± 3.2
					
**Tumor length ≥ 5 cm**^†^	116/199 (58.3%)	66/124 (53.2%)	25/43 (58.1%)	11/14 (78.6%)	14/18 (77.8%)
					
**Mean tumor diameter**^†^ (n = 81, cm, SD)	2.7 ± 1.3	2.6 ± 1.2	4.1 ± 1.2 *	2.5 ± 0.7	1.9 ± 0.1

**Histological type**^† ^(n = 271)					
SCC	183 (67.5%)	103 (70.1%)	45 (70.3%)	17 (56.7%)	18 (60.0%)
adenocarcinoma	83 (30.6%)	44 (29.9%)	18 (28.1%)	12 (40.0%)	9 (30.0%)
other	5 (1.8%)	0	1 (1.5%)	1 (3.3%)	3 (10.0%)

n: number of patients, cm: centimeter, SD: standard deviation, BSC: best supportive care,

SCC: squamous cell carcinoma, *: *p *< 0.05 curative treatment vs BSC,

^†^: not available for all patients

### Treatment of patients without visceral metastasis

In patients without visceral metastasis (n = 220), treatment with curative intent was performed in 151 patients (68.6%). (Table 3). Severe adverse effects were reported in 20.5% patients. Sixty-nine patients (31.4%) had no curative treatment as they were older (80 *versus *74.9 years, *p *< 0.01), had worse creatinine clearance (52.5 *versus *72.4, *p *< 0.01), worse albumin rate (31.4 *versus *37.7, *p *< 0.01), greater loss of weight (64.7% *versus *28.4% loss of weight ≥ 10%, *p *< 0.01), worse WHO performance status (20.4% *versus *79.3% WHO performance status at 0 or 1, *p *< 0.01) or worse Charlson score (18.2% *versus *38.5% Charlson score at 0, *p *< 0.01). In multivariate analysis, age (*p *= 0.02), loss of weight (*p*= 0.03), WHO performance status (*p *= 0.03) and Charlson score (*p *= 0.03) were significantly associated with BSC.

**Table 3:** Treatment and toxicity

	All patients (n = 282)	Patients without visceral metastasis (n = 220)		Patients with visceral metastasis (n = 62)	
		
		Curative treatment (n = 151)	BSC (n = 69)	Palliative treatment (n = 30)	BSC (n = 32)
**Dysphagia palliative treatment**					
naso-gastric tubes	15 (5.3%)	7 (4.6%)	4 (5.8%) *	3 (10.0%)	1 (3.1%) **
gastrostomy	11 (3.9%)	4 (2.6%)	6 (8.7%)	0	1 (3.1%)
endoscopic dilation	44 (15.6%)	19 (12.6%)	14 (20.3%)	5 (16.7%)	5 (3.1%)
oesophageal stent	63 (22.3%)	3 (2.0%)	37 (53.6%)	5 (16.7%)	18 (3.1%)
					
**Initial cancer treatment**					
Mucosectomy	6 (2.1%)	6 (4.0%)	-	-	-
PDT	18 (6.4%)	14 (9.3%)	3 (4.3%)	1 (3.3%)	-
Surgery	13 (4.6%)	13 (8.6%)	-	-	-
Chemoradiotherapy	119 (42.2%)	111 (73.5%)	-	-	-
Radiotherapy	8 (2.8%)	7 (4.6%)	-	-	-
Chemotherapy	22 (7.8%)	-	2 (2.9%)	20 (66.7%)	-
					
**Severe adverse effects**	48 (17.0%)	31 (20.5%)	4 (5.8%) *	10 (33.3%)	3 (9.4%) **

**Dysphagia evolution**^†^					
Regression at 2 months (n = 229)	139 (60.7%)	87 (64.0%)	25 (52.12%)	16 (55.2%)	11 (68.7%)
Recurrence (n = 103)	60 (58.2%)	35 (67.3%)	15 (68.2%)	7 (36.8%)	3 (30%)

n: number of patients, BSC: best supportive care, *: *p *< 0.05 curative treatment vs BSC,

**: *p *< 0.05 palliative treatment vs BSC, PDT: Phototherapy dynamic,

^†^: Many patients were not evaluable because they had died within 2 months

Twenty patients had a mucosectomy or PDT because the tumor was T1N0M0. Both treatments permitted 14 patients to achieve clinical complete response without major complications but 7 patients had a local recurrence.

Most of the patients benefited from CRT because in our institution all patients with stage III disease and/or surgical contradictions were treated by CRT. Selection was based on age (mean age of 74.3 ± 3.7), WHO performance status (79.8% WHO 0 or 1) and loss of weight (67.8% inferior to 10%). Twenty-seven patients (24.3%) experienced adverse effects ≥ grade 3, mainly vomiting and neutropenia. Sixty-four patients (57.6%) had a CCR and 27 (24.3%) had no recurrence during the follow-up. Local recurrence was 32.8% and metastasis occurrence was 27.9% for patients with CCR to CRT. Median overall survival was 17.5 ± 1.0 months and 2-year survival was 36.6%.

Thirteen patients underwent initial surgery and seven had salvage treatment due to no CCR to CRT or local recurrence. Mean age was 73.5 ± 2.6. No patients had adjuvant therapy. Four had severe post-operative complications (two patients died) and ten had tumor recurrence. During a follow-up of 27.5 months, local recurrence was 20% and metastasis occurrence was 35%. Median overall survival was 26.4 ± 7.9 months and 2-year survival was 58.8%.

### Treatment of patients with visceral metastasis

In patients with M1b disease (n = 62), 30 patients benefited from palliative treatment (table 3). Thirty-two patients had BSC as they were older (78.2 *versus *74.2 years, *p *< 0.01), and had worse WHO performance status (26.9% *versus *70% WHO performance status at 0 or 1, *p *< 0.01) and worse albumin rate (28.7 *versus *35.2, *p *< 0.01). None of these criteria were significant in multivariate analysis.

In patients treated by CT (n = 20), 7 had adverse effects ≥ grade 3. At first evaluation (3 months), seven patients had a stable disease or a partial response to CT.

### Best supportive care

On the whole, 133 patients (47.2%) initially had a palliative treatment for dysphagia (Table 3). After SEMS placement, 6 patients suffered a severe complication (aspiration pneumonia) with one death. After oesophageal dilatation without stent placement, one patient had pneumonia following treatment. One hundred and one patients had BSC alone and the majority had a SEMS placement for dysphagia palliation (54.4%).

### Outcome and overall survival

After 80 years, no patient without visceral metastasis (n = 50) benefited from surgery and only eight from CRT. Concerning patients with visceral metastasis older than 80 years (n = 13) only 1 patient had chemotherapy.

Dysphagia improvement with treatment was observed in 60.7% of the evaluated patients with no difference between groups (Table 3). After SEMS placement, few dysphagia recurrences were observed (n = 9/63).

**Figure 2:**
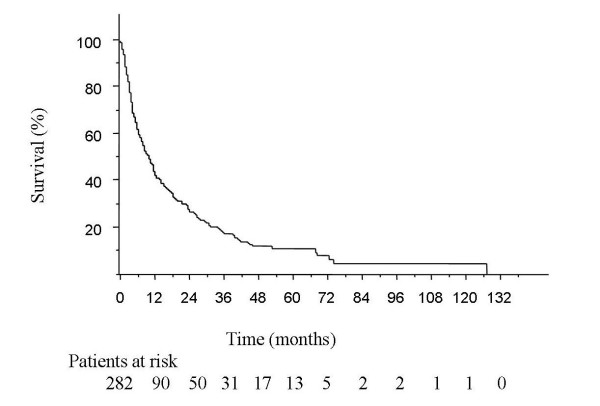
**Overall survival**. The median overall survival was 9.7 ± 1.0 months.

**Figure 3:**
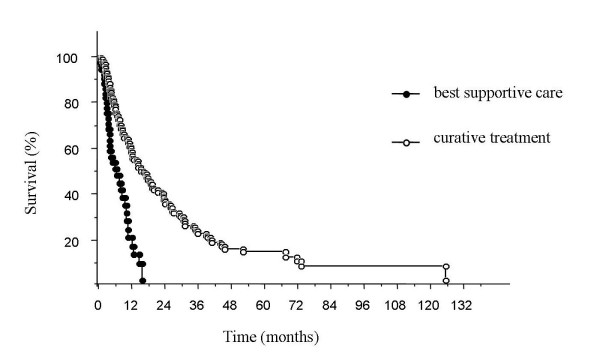
**Overall survival according to treatment in non-metastatic patients**. The median overall survival was 17.8 ± 1.5 months for patients with curative treatment and 5.5 ± 2.0 months for patients with best supportive care (BSC).

The median overall survival was 9.7 ± 1.0 months (Fig. 2) and specific survival was 11.5 ± 0.7 months (Table 4). Among non-metastatic patients, median overall survival was 17.8 ± 1.5 months in the curative treatment group versus 5.5 ± 2.0 months in the BSC group (Fig. 3). Predictive factors of overall survival in multivariate analysis were a WHO performance status < 2 (*p *< 0.01), initial weight loss < 10% (*p *= 0.01), an early tumoral stage (*p *< 0.01) and a carcinologic treatment (*p *< 0.01) but neither age nor comorbidities were considered factors (Table 5).

**Table 4:** Patient outcome and survival

	All patients (n = 282)	Patients without visceral metastasis (n = 220)		Patients with visceral metastasis (n = 62)	
		
		Curative treatment (n = 151)	BSC (n = 69)	Palliative treatment (n = 30)	BSC (n = 32)
**Overall survival **(month, SD)	9.7 ± 1.0	17.8 ± 1.5	5.5 ± 2.0 *	6.7 ± 2.1	1.8 ± 0.4 **
**Specific survival **(month, SD)	11.5 ± 0.7	23.2 ± 5.3	6.1 ± 2.5 *	6.7 ± 2.1	1.8 ± 0.4 **
**Deaths**	206 (73.0%)	113 (74.8%)	42 (60.9%)	24 (80.0%)	27 (84.4%)

**Causes of death**					
cancer	181 (87.9%)	91 (80.5%)	40 (95.2%)	24 (100%)	26 (96.3%)
treatment	5 (2.4%)	3 (2.6%)	1 (2.4%)	0	1 (3.7%)
other	20 (9.7%)	19 (16.8%)	1 (2.4%)	0	0

n: number of patients, SD: standard deviation, BSC: best supportive care, *: *p *< 0.05 curative treatment vs BSC, **: *p *< 0.05 palliative treatment vs BSC

**Table 5:** Predictive factors of overall survival in univariate and multivariate analysis

	Univariate analysis Median overall survival	Multivariate analysis HR [95% CI]
**Age ≥ 75 years**	ns	
		
**Sex**	ns	
		
**WHO performance status < 2**		
no	4.2 ± 0.2 *	1 [reference]
yes	17.8 ± 1.5	2.3 [1.6-3.4] *
		
**Initial weight loss < 10%**		
yes	6.3 ± 0.4 *	1 [reference]
no	17.5 ± 0.9	1.6 [1.1-2.3] *
		
**Charlson score ≤ 1**	ns	
		
**Histology**	ns	
		
**Tumor stage**		
IV	4.2 ± 0.2	1 [reference]
III	10.7 ± 2.3	1.7 [1.1-2.6] *
II	15.2 ± 2.8	1.6 [1.1-2.5] *
I	67.7 ± 7.0 *	4.5 [1.9-11.1] *
		
**Treatment**		
BSC	3.4 ± 0.3 *	1 [reference]
carcinologic treatment	14.3 ± 3.3	4.4 [2.4-8.1] *

HR: Hazard ratio, CI: confidence interval, BSC: best supportive care, *: *p *< 0.05, ns: not significant

## Discussion

This study highlights the clinical practice in our institution. The main results were: (i) the prognosis of oesophageal cancer in elderly patients remains poor (median overall survival at 10 months) (ii) among patients without metastasis, 68.6% underwent a treatment with curative intent but among patients with a M1b disease the majority had BSC (51.6%) (iii) selected patients with good performance status, good nutritional status and without major comorbidities were able to benefit from curative treatment or chemotherapy without major adverse events. Considering the few serious adverse events, the criteria used to select patient's treatment were appropriated and could be used in clinical practice. Although treatment of elderly patients with EC may be associated with an appreciable morbidity, at 75 years of age considerable life expectancy remains (above 10 years) [17]. Then, elderly patients with EC may undergo cancer treatment, and exclusion should be decided, as for younger subjects, on an individual basis. Nevertheless in our series, after 80 years of age few patients underwent an aggressive cancer treatment such as surgery, chemotherapy or chemoradiotherapy.

Endoscopic resection and PDT are the treatments of choice in most patients with mucosal EC [18,19] but may also be used for T1N0M0 oesophageal carcinoma with submucosal invasion as an alternative to surgery in elderly patients with comorbidities [18]. The complete response rate from was 40% to 80% [15,18,19]. In our series, mucosectomy and PDT were an effective treatment of early EC in elderly patients with 70% (n = 14/20) of complete response but 50% (n = 7/14) of local recurrence. Surgical approach in patients older than 70 years with oesophageal cancer remains debated because of potentially higher post-operative complications [5,20,21]. As in our study, median survival after esophagectomy remains poor in elderly patients, ranging from 6 to 27 months with post-operative mortality ranging from 4.7% to 7.2% [22]. Average survival almost approached the expected lifespan of a younger cohort.

Definitive CRT is considered a feasible nonsurgical treatment in patients with a locally advanced oesophageal cancer and approximately a 50% to 65% CCR rate, 17 to 26 months of median overall survival and 30% to 40% 2-year survival rate [8,9,16]. Recently, two large randomized trials investigated the efficacy of CRT, in the Stahl *et al *study patients up to 70 years were excluded and in Bedenne *et al*'s study there was no stratification according to age of patients [8,9]. Interestingly, our results in elderly patients were relatively close to those reported in these studies. Anderson et al. and our group reported significant results of CRT in elderly patients in accordance with those in younger patients, which suggested that this treatment can be proposed with curative intent [10,11].

Chemotherapy in metastatic EC has poor efficacy and has not been validated through large randomized trials; moreover, there is no data reported in the literature on chemotherapy tolerance and efficacy in elderly patients with EC. In metastatic patients with median age 55-65 years, chemotherapy based on 5FU and cisplatin showed a 20 to 40% response rate with a median overall survival approximately 8-13 months. Adverse events grade ≥ 3 have been reported in 25-60% of cases [23]. In our series, twenty patients had a palliative chemotherapy for metastatic EC, seven experienced adverse effects ≥ grade 3 (35%) and seven (35%) had disease control at 3 months. Moreover, 5FU and cisplatin tolerance were reported as the same in the elderly and younger patients for advanced esophago-gastric cancer [24]. These results are in agreement with those in younger patients and underline the possible usefulness of chemotherapy in selected elderly patients.

In France, approximately 20% of patients with EC receive BSC alone and in the USA, 16% of patients over 65 years receive BSC alone [4]. These results of cancer registry were probably underestimated. In our study, 35.8% (n = 101/282) of patients had BSC without any specific cancer treatment.

To our knowledge, no previous study has investigated therapeutic decisions in elderly patients with EC. In our study, the main factors which influenced treatment strategy were age, WHO performance status, nutritional status and comorbidity. Using these criteria, few patients experienced severe treatment**-**related complications. An oncogeriatric assessment before treatment could be an important tool for oncologists when making treatment decisions, and it should be evaluated in EC. A clinical score might be of appreciable help in clinical decisions. According to our results, several criteria including age > 75 years, loss of weight > 10%, WHO performance status > 1 and Charlson score > 1 are contradictions for aggressive treatment. These criteria need to be validated by prospective analysis.

As reported in the literature, median overall survival is poor (10 months) in elderly patients with an EC and worse than in younger patients [4]. The main reported predictive factors of overall survival were WHO performance status, nutritional status and TNM stage [25,26]. No major difference was found in our multivariate analysis among elderly patients. Our group has previously shown that baseline nutritional status is predictive of response to treatment and survival in patients treated by definitive CRT for a locally advanced oesophageal cancer [26].

The major limitation of our study was that the retrospective analysis may have been based on incomplete medical records. Nevertheless, the majority of non-available data were due to the patient's death. Others limitations are experience in a single institution, the small sample size and the lack of pre-defined factors determining treatment decisions, which were based only on evaluations by the referral doctor and members of multi-disciplinary team. It should nonetheless be recalled that the aim of the study was to retrospectively identify the parameters to be associated with the key therapeutic decision.

## Conclusions

In conclusion, for the management of elderly patients with an EC, in addition to age it is important to evaluate nutritional status, performance status and comorbidity before deciding therapeutic strategy. Our study suggests that "selected elderly patients" with these criteria should benefit from curative treatment because there exists is no major related toxicity treatment and similar treatment efficacy. Since our study is a retrospective analysis, these data warrant confirmation in further series.

## Authors' contributions section

DT made contributions to design, acquisition, analysis and interpretation of data, and was involved in drafting the manuscript. HH participated in the acquisition and analysis of data and in drafting the manuscript. MS participated in the interpretation of data and in drafting the manuscript. FDF participated in the interpretation of data and in drafting the manuscript. MA participated in the acquisition of data and in drafting the manuscript. BP participated in the analysis of data and in drafting the manuscript. PM participated in the interpretation of data and in its design and coordination. All authors read and approved the final manuscript.

## Conflict of interests statement

The authors declare that they have no competing interests.

## Source(s) of support

None

## Pre-publication history

The pre-publication history for this paper can be accessed here:

http://www.biomedcentral.com/1471-2407/10/510/prepub
